# Radiation Protection Device Composite of Epoxy Resin and Iodine Contrast Media for Low-Dose Radiation Protection in Diagnostic Radiology

**DOI:** 10.3390/polym15020430

**Published:** 2023-01-13

**Authors:** Nutthapong Moonkum, Chalermchai Pilapong, Krai Daowtak, Gunjanaporn Tochaikul

**Affiliations:** 1 Faculty of Radiological Technology, Rangsit University, Rangsit 12000, Thailand; 2 Department of Radiologic Technology, Faculty of Associated Medical Sciences, Chiang Mai University, Chiang Mai 50200, Thailand; 3 Department of Medical Technology, Faculty of Allied Health Sciences, Naresuan University, Phitsanulok 65000, Thailand

**Keywords:** iodine contrast media, radiation shielding, epoxy resin

## Abstract

Radiation protection in radiology is important because radiation may cause harm to the human body. The equipment for radiation protection is essential to ensure safe operations. Currently, there is widespread research on lead-free radiation shielding material. The aim of this research was to study lead-free material containing epoxy and iodine contrast media that was easy to form, low in cost, and environmentally friendly. The results showed that 2-cm material thickness with a concentration of 20% iodine had the greatest properties of radiation attenuate in the peak potential applied at technique 60–120 kVp, but the structure and strength of the shielding materials were decreased in accordance with increasing concentrations of iodine contrast media. It can be concluded that the lead-free epoxy radiation-shielding materials are able to absorb radiation at energy levels of 60–120 kVp. However, with improvement on homogeneity in the future, it could be used as a refractory shielding material in the radiology department.

## 1. Introduction

Medical radiation is used in many medical imaging procedures and radiographs to help determine the cause of symptoms and necessary radiation for the treatment of various diseases [1]. The use of radiation is very useful in medicine but if used carelessly without protection, it would be dangerous to humans and other living things [2]. Unnecessary exposure to radiation can cause molecular changes at the cellular level and can cause damage to DNA or cancer [3]. Radiation protection is the protection of people from the harmful effects of exposure to ionizing radiation [4]. The radiation exposure may come from radiation sources outside the human body or from medical radiation [5], including various contaminants caused by radioactive substances [6,7]. In general, the radiology department must have equipment for radiation shields or a means to attenuate radiation from causing harm to the radiological technologist or radiologist [8]. Lead is a radiation-shielding device with a high atomic number [9]. It has good radiation-protection properties, but the disadvantage is that it is heavy and also highly toxic [10]. For these reasons, studies have been carried out, including inventing materials that can replace lead and provide radiation protection.

Previous studies have found that new lead-free shielding materials have radiation-shielding properties by using components with high-atomic-number substances such as tungsten trioxide (WO_3_) [11], barium sulphate (BaSO_4_) [12], bismuth oxide (Bi_2_O_3_) [13,14], and molybdenum trioxide (MoO_3_) [15]. For example, the shielding properties from epoxy resin with nano-WO_3_ for reduction of gamma rays from Cs-137 and Co-60 showed that the dose reduction was 46% and 34% for Cs-137 and Co-60 at 40% concentration in all samples [16]. The properties of reducing photon energies of 22.16–59.54 keV of CeO_2_ and Er_2_O_3_ in the BS samples erbium oxide and cerium oxide in borosilicate glasses increased radiation absorption with an increasing concentration of components [17]. The performance of the bismuth titanate (Bi_4_Ti_3_O_12_) nanoparticle (0–65 wt%) with epoxy composite for radiation attenuation had efficiencies of 97% and 95% at 80 and 100 kVp, respectively [18]. Moreover, the study by Monte Carlo simulations and theoretical calculations also found that the various borate glasses including Bi, V, Fe, and Cd have the properties of photon attenuation for nuclear shielding [19] and the shielding performances of five different borosilicate-based glassed can shield against potential radiation damage to environmental health [20] and also found a glass of composition TeO_2_, CdO, PbO, and Bi_2_O_3_ have the efficiency of gamma-ray shielding [21]. However, there is still developmental research of contrast medias in radiography to study radiation-shielding potency. 

In previous research, it was found by Monte Carlo simulation that the shielding ability of barium sulfate and iodine contrast media is impressive; they greatly absorb radiation in the energy region of 120 kVp, and iodine contrast media has better efficiency in terms of radiation shielding than barium sulfate [22]. Additionally, the iodine contrast media had potency of radiation shielding for absorbing scatter ray by using an acrylic sheet filled with iodine contrast substance to replace general lead glass [23]. However, the potency properties and material forming of iodine contrast media lack data about efficiency for attenuation of radiation. In this work, we aimed to study the radiation-shielding properties of our radiation-shielding material, which a lead-free and environmentally friendly mixed epoxy resin and iodine contrast media in different proportions and compare the results to commercial lead shielding at the applied voltages of 60–120 kVp.

## 2. Materials and Methods

### 2.1. Preparation of Epoxy Resin Material Radiation Shielding

Epoxy resin was purchased from Resin lab with 1.21 g/cm^3^ density (Thailand), and iodine contrast media (370 mg/mL) was purchased from Bayer (Germany). The materials were mixed for 10 min in a 500-mL beaker with four formulations (%*w/w*) as shown in Table 1, and then the material was added to the block (10 × 10 × 2 cm^3^) and cured at room temperature for 72 h by forming three pieces per ratio. This project was approved by the ethical review committee for research in human subjects, Faculty of Radiological Technology, Rangsit University (COA. No. RSUERB2022-068).

**Figure 1:**
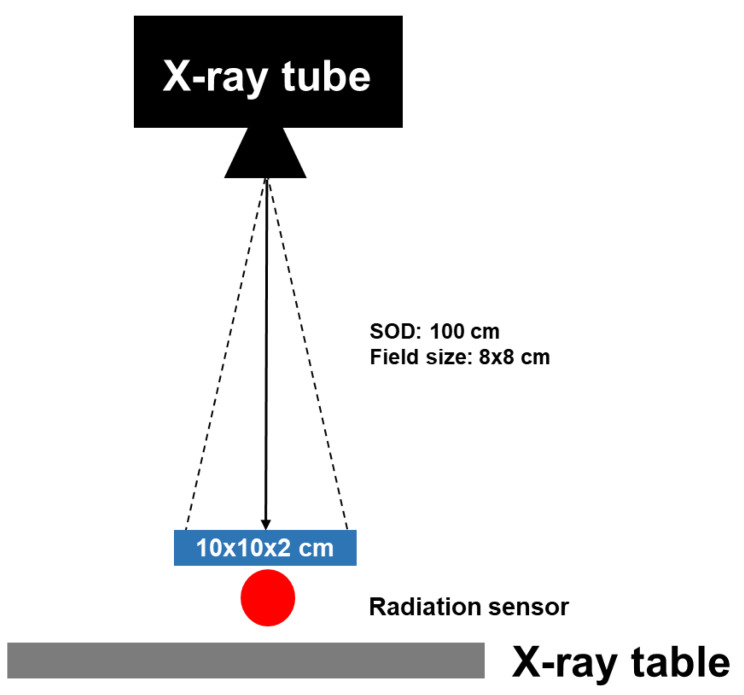
Schematic view of the X-ray radiation shielding tests.

The epoxy resin shielding materials were irradiated by X-ray (FDR smart X/Fujifilm) at a distance of 100 cm from the X-ray source to material and a field size of 8 × 8 cm^2^ and radiation dose was measured by Radcal Accu Gold in Figure 1. 

### 2.2. The Efficiency of Epoxy Resin Shielding Material Measurements 

The radiation absorption properties were measured from the X-ray with the applied energy voltages from 60–120 kVp irradiated by general X-ray (FDR smart X/Fujifilm). The absorption properties of epoxy resin shielding material were described by Equation (1) [24]:

(1) $$\frac{\left(Absorption\;dose\;without\;shielding-Absroption\;dose\;with\;shielding\right)}{Absroption\;dose\;without\;shielding}\times 100$$

### 2.3. Materials Characterization of Epoxy Resin Shielding Material

The shielding material was measured in grams (g), and the morphology was characterized by JEOL JSM-IT300 scanning electron microscope. For strength measurement, a tensile strength test (1TK-10TX, BEMAX, Osaka, Japan) loading at 1–10 ton and X-ray fluorescence spectrometer–XRF (BRUKER S8 TIGER) analyzed the iodine contrast media in the epoxy resin shielding material

## 3. Results

### 3.1. The Structural Characterization of Epoxy Resin Shielding Material

**Figure 2:**
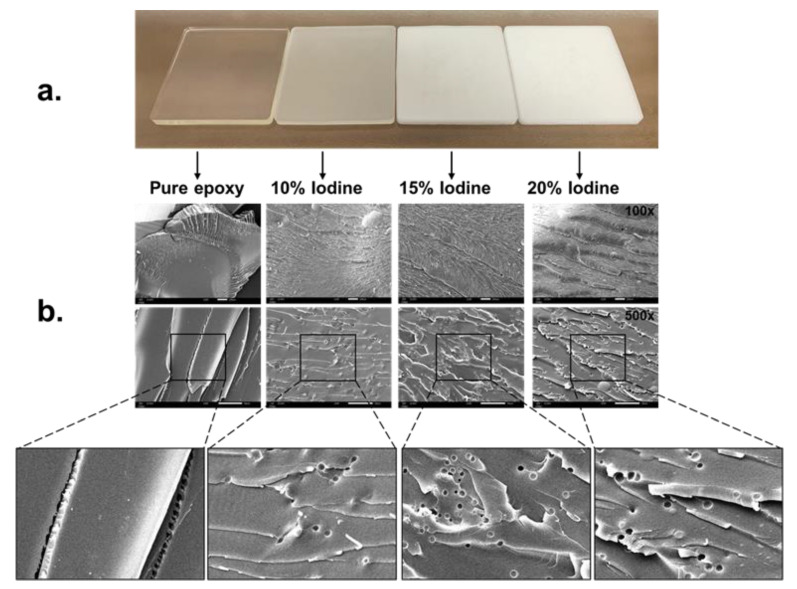
(**a**) The characteristic of epoxy resin shielding material. (**b**) The homogeneity of epoxy resin with composite of iodine contrast media were analyzed by scanning electron microscopy at 100× and 500×.

The addition of iodine contrast media had no effect on the strength of the shielding material and only affected opacity (see Table 2 and Figure 2).

Homogeneity of the radiation shielding materials were analyzed by using scanning electron microscope (SEM) and the sample materials were broken into small pieces before examination. In Figure 2, the results showed that the distribution of pure epoxy resin material was highly homogenous, but when mixing composite of iodine contrast media, it was found that the shielding material began to decrease in order of homogeneity with the increasing composite of iodine contrast media and air bubble formation. These results showed that the morphology of shielding material may indicate that the homogeneity decreases with the increase of iodine contrast media.

### 3.2. Radiation-Shielding Properties 

**Figure 3:**
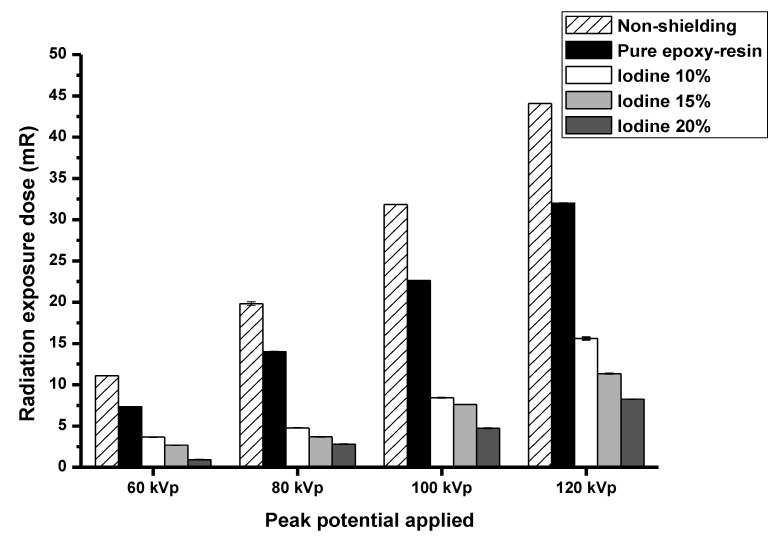
The X-ray dose measurement at different kVp.

The radiation-shielding properties were analyzed from exposure technique (60, 80, 100, and 120 kVp). The epoxy resin containing 20% iodine contrast media showed the greatest protection from the radiation doses (Figure 3).

### 3.3. Radiation Absorption Properties 

**Figure 4:**
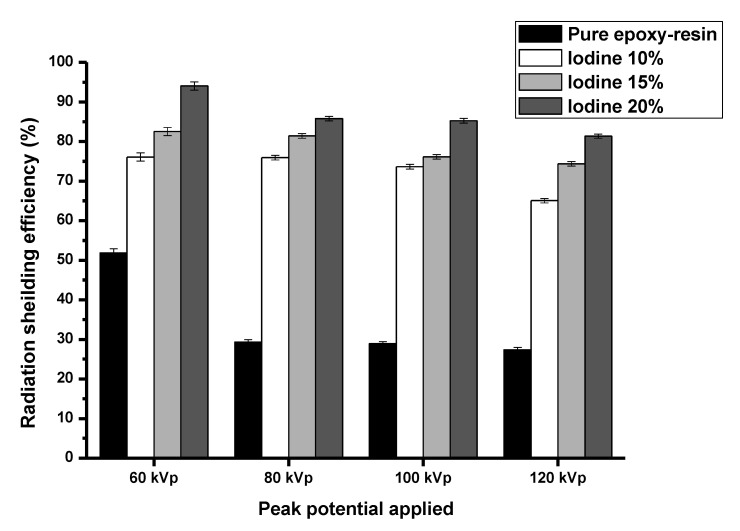
The radiation absorption at 60, 80, 100, and 120 kVp.

In Figure 4, the radiation absorption was calculated from Equation (1). The radiation absorption from pure epoxy shielding material with concentrations of iodine contrast media composite (10%, 15%, and 20%) were 27.39–51.86%, 65.06–76.06%, 74.36–82.50% and 81.35–94.01%, respectively. The results show that the radiation absorption increases with increasing concentration of iodine contrast media and that the epoxy shielding material with 20% iodine contrast media had the highest radiation absorption properties.

**Figure 5:**
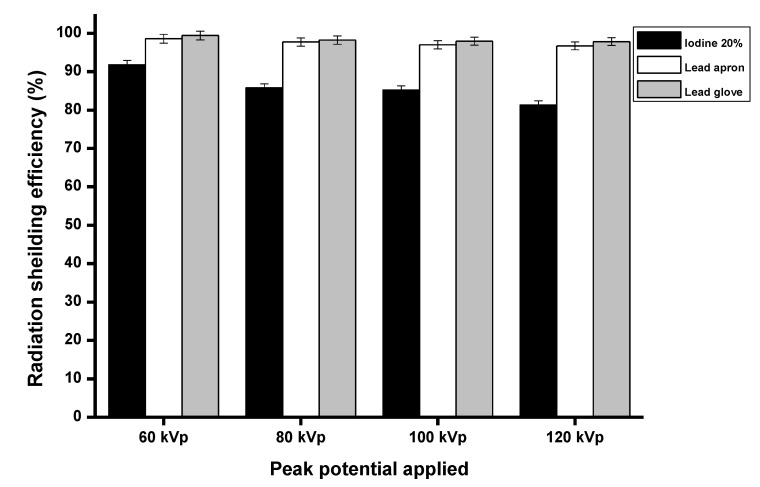
The radiation-absorption properties between the shielding material of 20% iodine contrast media concentration with lead apron and lead glove.

Therefore, we chose the epoxy resin with 20% iodine contrast media concentration to compare with lead aprons (Uniray universal, thickness: 0.50 mm Pb) and lead gloves (ProtecX, thickness: 0.50 mm Pb). The results indicated that the radiation absorption in epoxy shielding materials is less than that of lead aprons and lead gloves, as seen in Figure 5.

**Figure 6:**
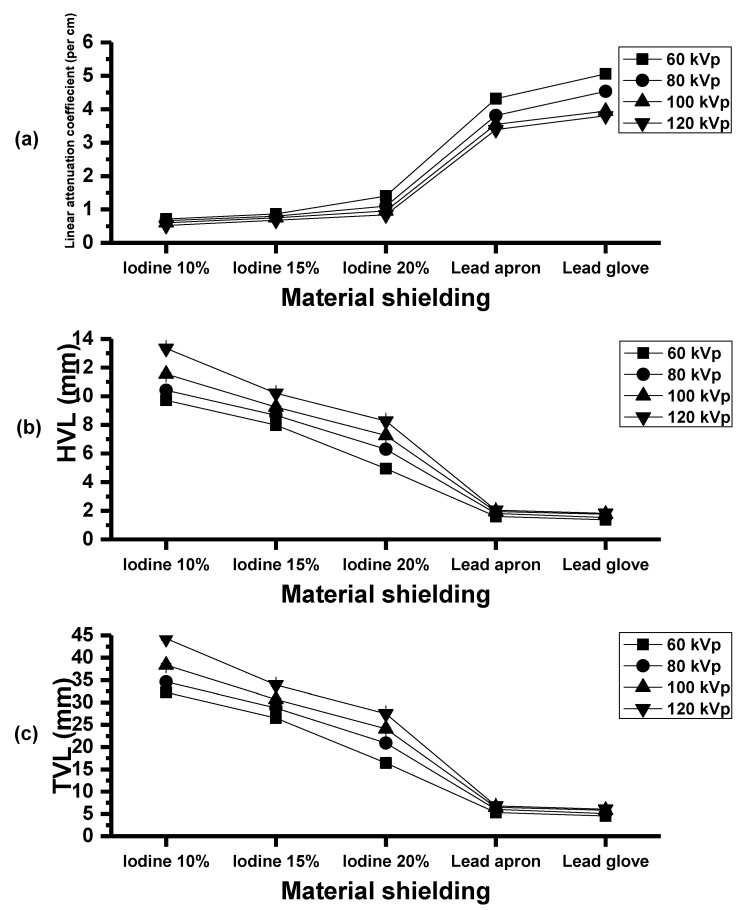
(**a**) Linear attenuation coefficient of the epoxy shielding material. (**b**) Half-value layer and (**c**) tenth-value layer of epoxy shielding material.

In addition, the linear attenuation coefficient of epoxy shielding material with iodine contrast media composites were increased and were highest in composite of 20% iodine contrast media (Figure 6a). The half-value layer (HVLs) and tenth-value layer (TVL) were decreased and lowest in composite of 20% iodine contrast media (Figure 6b,c).

These results showed the properties of iodine contrast media composites when mixing with epoxy resin for radiation protection. The high concentration of iodine contrast media in the material can reduce the radiation dose from exposure of 60–120 kVp. However, the homogeneity of the radiation-shielding material may decrease with increasing concentrations of iodine contrast media.

### 3.4. Tensile Strength, Maximum Force (N) and X-ray Fluorescence (XRF)

**Figure 7:**
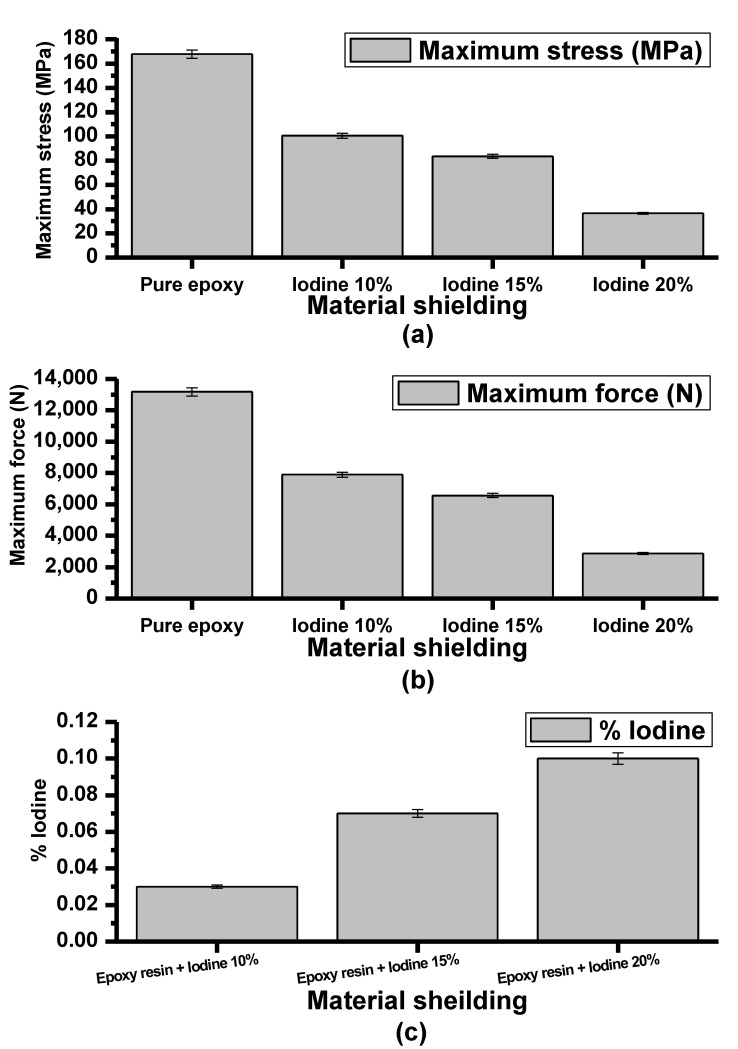
Comparison of (**a**) maximum stress (MPa), (**b**) maximum force (N), and (**c**) X-ray fluorescence.

Figure 7a shows the maximum stress (MPa) of pure epoxy material and epoxy material with iodine contrast media composite as 167.76, 100.57, 83.61 and 36.51 MPa, respectively. Maximum force (N) also decreased with increasing concentrations of iodine contrast media (Figure 7b): 13,176.05, 7899.03, 6567.03, and 2868.01 N, respectively.

Figure 7c shows the X-ray fluorescence (XRF) analysis of the iodine contrast media in epoxy resin shielding material. From Figure 7c, the concentration of iodine contrast media was found in the composition according to the proportional mixing that was formed. 

These findings demonstrate that epoxy shielding material has the properties of radiation absorption in the exposure range of 60–120 kVp, 4 mAs. In addition, the increasing radiation absorption is correlated with increasing the composite of iodine contrast media in epoxy resin but the structure of materials was decreased in alignment with increased concentration of iodine contrast media.

## 4. Discussion

Over the past few years, there has been interest in researching radiation shielding, the most common method of which is lead shielding, which protects patients and workers from unnecessary medical radiation [25]. This research has created radiation-shielding materials that are composites of epoxy resin and iodine contrast media, which are lead-free and strong. Our results have shown that this material is strong and able to withstand pressure. Additional important characteristics are its resistance to chemicals [26], heat resistance [27], adhesion to a variety of substrates [28], high tensile nature [29], and high electrical insulation and retention properties [30], including corrosion resistance [31] and high atomic number for greatly increased photon attenuation [32]. Epoxy resin is a synthetic polymer used as a composite radiation-shielding material [33] with other high-atomic-number materials, such as tungsten oxide (WO_3_) [34], gadolinium oxide (Gd_2_O_3_) [35], barium sulphate (BaSO_4_) [12], bismuth oxide (Bi_2_O_3_) [14], etc. Our results were in accordance with the radiation-shielding properties of epoxy resins with high-atomic-number composites [36,37]. In this research, the epoxy resin composite of iodine contrast media had good radiation protection and compared favorably with commercial lead shielding. The radiation absorption was slightly less than that of the lead apron and lead gloves, but the impact toughness and hardness of epoxy resin shielding materials were higher. This composite had the ability to attenuate X-ray at energy levels of 60–120 kVp as 92–82%. 

In this research, the concentration of iodine contrast media less than 20% was chosen because it may result in the material being unable to form (result not shown). An additional deciding factor was that the dispersion of iodine contrast media in the epoxy resin shielding may affect the chosen XRF analytical method, as EDX cannot be detected with low concentrations of shielding. Moreover, the epoxy sheet material retained its strength and the physical properties include low cost, environmental friendliness, and X-ray absorption similar to commercial lead shielding. Although there is quite a lot of research on material shielding from epoxy resin with other composites from radiation protection [38,39], we used materials that can be readily available in diagnostic radiology, especially iodine contrast media, which can be easily found, is inexpensive to procure, and could be applied for X-ray-shielded walls in the future. 

The construction material Is strong when analyzed by scanning electron microscopy (SEM) and had high tensile strength. However, the shielding material structure becomes less homogeneous and the tensile strength is reduced when the composite of iodine contrast media was added in epoxy resin material. This may be caused by an incomplete mixing process and generation of air bubbles during the experiment. These results are consistent with previous studies, showing that when various ingredients were added, the epoxy would decrease in strength. [40,41]. Refinement of the molding process should be addressed and this initial study focused on fabricating the material and measured the radiation attenuation efficiency. It was found that a low concentration (<20%) of iodine contrast media to maintain the material shape. In the future, we will develop epoxy resin shielding materials with a high concentration of iodine contrast media and study together with Monte Carlo methods.

## 5. Conclusions

In conclusion, this research aimed to study the radiation absorption properties of epoxy resin and iodine contrast media including the development of a radiation shielding material for diagnostic radiology that is easy to form and lead free. The radiation shielding materials from epoxy resin and iodine contrast media are highly durable and can actually prevent radiation-related complications. Moreover, the radiation-shielding material is nontoxic and environmentally friendly. However, in the future it may be necessary to develop epoxy resin shielding materials with a high composite of iodine contrast media of more than 20% in order to be able to shield a variety of energy radiation.

## Figures and Tables

**Table 1:** Epoxy resin with iodine contrast media in different proportions (*v*/*v*).

Formula Number	Iodine Contrast Media (%)	Epoxy Resin (%)
No.1	-	100
No.2	10	90
No.3	15	85
No.4	20	80

**Table 2:** The characterization of the material.

Shielding Material: Iodine Contrast Media (%)	Weight (g)	Density (g/cm^3^)	Light Transmission
Pure epoxy resin	174	1.21	Transparent
10% Iodine contrast media	180	1.23	Opaque
15% Iodine contrast media	184	1.24	Opaque
20% Iodine contrast media	188	1.25	Opaque

## Data Availability

Not applicable.
